# Multi-Proteomic Insights into Lysine Propionylation and Malonylation Remodeling in PRRSV-Infected Porcine Lungs

**DOI:** 10.3390/vetsci13090931

**Published:** 2026-09-09

**Authors:** Yue Feng, Dake Chen, Houchun Liu, Mu Qiao, Zhong Xu, Shuqi Mei, Xianwen Peng, Junjing Wu

**Affiliations:** 1 Key Laboratory of Animal Embryo Engineering and Molecular Breeding of Hubei Province, Institute of Animal Sciences and Veterinary Medicine, Hubei Academy of Agricultural Sciences, Wuhan 430064, China; 2 Hubei Hongshan Laboratory, Wuhan 430070, China

**Keywords:** PRRSV, propionylation, malonylation, multi-proteomics

## Abstract

Porcine reproductive and respiratory syndrome virus is a major pathogen that causes massive economic losses in the global swine industry. Lysine propionylation and malonylation are metabolism-sensitive post-translational modifications, yet their coordinated regulatory roles during PRRSV pulmonary infection remain unknown. This study combined quantitative proteomics, propionylome and malonylome to characterize host molecular alterations between healthy and PRRSV-infected porcine lung tissues (three piglets per group). Quantitative proteomics identified 1467 significantly downregulated proteins and only 129 upregulated proteins, indicating a profound host protein shutoff during PRRSV pulmonary infection. The two acyl modifications displayed opposite regulatory patterns independent of global protein expression changes: 51 propionylation sites (24 proteins) were upregulated with only 1 downregulated, while 37 malonylation sites (30 proteins) were downregulated and only 5 upregulated. Functional enrichment and PPI network analysis revealed clear functional divergence: hyper-propionylated hub proteins were exclusively enriched in mitochondrial energy metabolism pathways, whereas hypo-malonylated core proteins were mainly involved in lipid metabolism and cell fate regulation. Integrated multi-omics analysis confirmed that protein expression and lysine acylation constitute two coordinated but independent regulatory layers, with histone H4 identified as a candidate target of competitive propionylation/malonylation. This work reports the comprehensive landscape of lysine propionylation and malonylation upon PRRSV infection, reveals a dual-PTM remodeling strategy for viral hijacking of host homeostasis, and provides candidate targets for future functional investigation and antiviral development.

## Simple Summary

Porcine reproductive and respiratory syndrome is a widespread disease in pigs that causes major financial losses for pig farmers around the world. Better understanding of how this disease harms pig lungs can help us find new ways to protect pigs. In this study, we examined lung tissues from healthy and infected pigs to explore protein and post-translational modification changes triggered by the infection. The results revealed that the PRRSV-infection greatly suppressed protein expression and changed propinylation and malonylation levels on cell proteins which showed the opposite pattern. One set of modification increased and affected energy-producing structures inside cells, while the other set decreased and influenced fat processing and cell survival. Our findings reveal how the disease agent disturbs normal pig cell functions. These new insights offer potential clues for developing future protective tools to reduce economic harm within the pig-raising industry.

## 1. Introduction

Porcine reproductive and respiratory syndrome (PRRS) is a highly contagious swine disease caused by the PRRS virus (PRRSV), which has inflicted substantial economic losses on the global pig industry. PRRSV infection causes reproductive failure in sows, respiratory illness in piglets, and high mortality rates, severely hampering sustainable swine production [1]. Although extensive research has been conducted on PRRSV epidemiology, virology, and host immune responses [2,3,4,5,6,7], the molecular mechanisms of post-translational modification (PTM) underlying virus–host interactions remain incompletely understood, which has hindered the development of effective prevention and control strategies.

Proteomic analysis alone cannot fully elucidate viral pathogenesis; integration of post-translational modifications could provide another mechanistic insight. Post-translational modifications (PTMs) of host proteins play crucial roles in viral infection [8,9,10,11,12,13,14,15]. PTMs refer to covalent modifications—such as phosphorylation, acetylation, ubiquitination, malonylation, and others—that occur after translation and regulate protein activity, stability, subcellular localization, and interactions with other biomacromolecules [16,17]. These modifications are integral to numerous cellular processes, including signal transduction, gene expression and protein folding and degradation [18,19,20]. Growing evidence suggests that PRRSV intricately modulates host PTMs to facilitate its own replication and spread. Concurrently, host cells employ PTMs to counteract viral invasion, for instance, by degrading viral proteins via the ubiquitin–proteasome pathway [21] or activating antiviral signaling through phosphorylation [22]. Therefore, elucidating the alterations in host PTMs during PRRSV infection may enhance our understanding of the molecular basis of virus–host interactions and identify potential targets for antiviral intervention.

In recent years, novel types of acylation modifications, such as propionylation and malonylation, have gained increasing attention. Propionylation involves the addition of a propionyl group (-COCH_2_CH_3_) [23] to lysine residues, whereas malonylation entails the attachment of a malonyl group (-COCH_2_COO^−^) [24]. These modifications can alter the charge and conformation of target proteins, thereby influencing their activity, stability, and protein interactions [25]. Studies have indicated that both propionylation and malonylation are metabolism-sensitive acylations, involved in mitochondrial energy and lipid metabolic regulation and inflammatory responses [26,27,28,29], both being processes that are central to PRRSV pathogenesis. Furthermore, PRRSV infection has been shown to induce autophagy in host cells [30], a process regulated the cell metabolism. These suggest that propionylation and malonylation may similarly be exploited by the virus.

PRRSV infection markedly reshapes the PTM landscape of host cells. Existing proteomic studies on PRRSV have primarily focused on phosphorylation, ubiquitination, glycosylation, and palmitoylation [31,32,33], while the roles of acylation modifications—particularly malonylation and propionylation—in viral infection remain largely unexplored. Although the potential roles of propionylation and malonylation in PRRSV infection are suggested, further studies are necessary to confirm their functions. Thus, investigating changes in these PTMs during infection could provide new insights into the mechanisms of PRRSV pathogenesis.

In this study, we applied label-free quantitative proteomics to lung tissue samples from PRRSV-infected pigs to systematically examine virus-induced alterations in host protein propionylation and malonylation. By integrating quantitative proteome and PTM omics data, we report the comprehensive landscape of lysine propionylation and malonylation upon PRRSV infection, reveal a dual-PTM remodeling strategy for viral hijacking of host homeostasis, and provide candidate molecular targets for future functional investigation.

## 2. Materials and Methods

### 2.1. Experimental Design and Sample Collection

All animal samples used in this experiment were collected under the supervision and guidance of the Experimental Animal Ethics Committee of Institute of Animal Sciences and Veterinary Medicine, Hubei Academy of Agricultural Sciences (2026CS014). Six 5-week-old piglets free of African swine fever virus, classical swine fever virus, pseudorabies virus, porcine circovirus type 2, and PRRSV were randomly divided into two groups: healthy group and infected group. Piglets in the infected groups were inoculated with 4 × 10^5^ TCID_50_ of PRRSV (strain WUH3, provided by Prof. Ao Zhou from Wuhan Polytechnic University) via a combination of intranasal (2 mL) and intramuscular (2 mL) routes. Healthy group piglets received an equal volume of RPMI 1640 medium (Gibco, Grand Island, NY, USA) via the same routes. At 21 days post-infection, all piglets were euthanized for necropsy, and lung samples were collected for further analysis.

### 2.2. Verification of PRRSV Infection by RT-PCR and Gel Electrophoresis

Prior to proteomic analysis, the PRRSV infection status of all lung tissue samples was confirmed by reverse transcription–polymerase chain reaction (RT-PCR) followed by agarose gel electrophoresis. Total RNA was extracted from approximately 30 mg of each lung tissue sample using the RNA extraction kit (Tiangen, Beijing, China). The RNA was then reverse-transcribed into complementary DNA using the HiScript II Reverse Transcriptase kit (Vazyme, Nanjing, China). Subsequently, conventional PCR was performed using specific primers targeting the PRRSV gene (PRRSV ORF7-F: 5′-GTTTGTGCTTGCTAGGCCG-3′; PRRSV ORF7-R: 5′-CTGCCACCCAACACGAGG-3′). Primers for porcine β-actin (β-actin-F-: 5′-CCAGGTCATCACCATCGG-3′; β-actin-R: 5′-CCGTGTTGGCGTAGAGGT-3′) were used as an internal control to assess RNA quality and cDNA synthesis efficiency. The PCR products were separated on a 2% agarose gel stained with GoldView (Yuanye, Shanghai, China). The gel was then directly imaged using a gel documentation system.

### 2.3. Protein Extraction and Digestion

Tissues were pulverized in liquid nitrogen and lysed by sonication in a lysis buffer containing 8 M urea and 1% protease inhibitor cocktail. Supernatants were collected after centrifugation, and protein concentration was determined using a bicinchoninic acid assay. Proteins were acetone-precipitated, and equal amounts of protein were resuspended in 200 mM triethylammonium bicarbonate buffer, followed by digestion with trypsin (1:50, *w*/*w*) at 37 °C overnight.

### 2.4. Enrichment of Modified Peptides

To enrich peptides bearing propionylation and malonylation modifications, the tryptic digests were incubated with specific anti-propionyl or anti-malonyl lysine antibody conjugated agarose beads (PTM-202/PTM-904). The resin was then washed to remove non-specifically bound peptides. Finally, the captured peptides were eluted, desalted using C18 ZipTip microcolumns (Merck, Carrigtwohill, Ireland), and lyophilized for LC-MS/MS analysis.

### 2.5. Liquid Chromatography–Tandem Mass Spectrometry (LC-MS/MS) Analysis

Peptide separation was performed using a nanoElute ultra-high performance liquid chromatography system (Bruker Daltonics, Shanghai, China). The mobile phases consisted of solvent A (0.1% formic acid in water with 2% acetonitrile) and solvent B (0.1% formic acid in acetonitrile). The separated peptides were subsequently analyzed on a timsTOF Pro mass spectrometer (Bruker Daltonics) operated in parallel accumulation–serial fragmentation data acquisition mode.

### 2.6. Integrated Proteomic and PTM Omics Analysis

Mass spectrometry data from the quantitative proteome and PTM-enriched samples were collectively analyzed using MaxQuant (version 1.6.15.0) against the Sus scrofa reference proteome. Search parameters included carbamidomethylation of cysteine as a fixed modification; protein N-terminal acetylation, methionine oxidation, and lysine propionylation or malonylation were set as variable modifications for the respective datasets. The false discovery rate was controlled at 1% for both proteins and peptide–spectrum matches. For quantitation of proteins and PTM sites, the signal intensity values of modification sites or proteins across different samples are subjected to centering transformation to obtain the relative quantitative values of modification sites or proteins in different samples. Then, the relative quantitative value of a modification site is divided by the relative quantitative value of its corresponding protein to eliminate the influence of protein expression on modification abundance. We identified differentially expressed proteins and modified sites by applying a threshold of |fold change| > 1.5 and a *p*-value < 0.05. Functional enrichment analysis for Gene Ontology (GO) terms and KEGG pathways was performed using Fisher’s exact test. Protein–protein interaction (PPI) networks were constructed using the STRING database (version 12.0), with interactions filtered at a confidence score > 0.9 for the quantitative proteome and propionylome, and >0.7 for the malonylome.

## 3. Results

### 3.1. Proteome and Post-Translational Modification Proteome Atlas of PRRSV-Infected Porcine Lung

**Figure 1:**
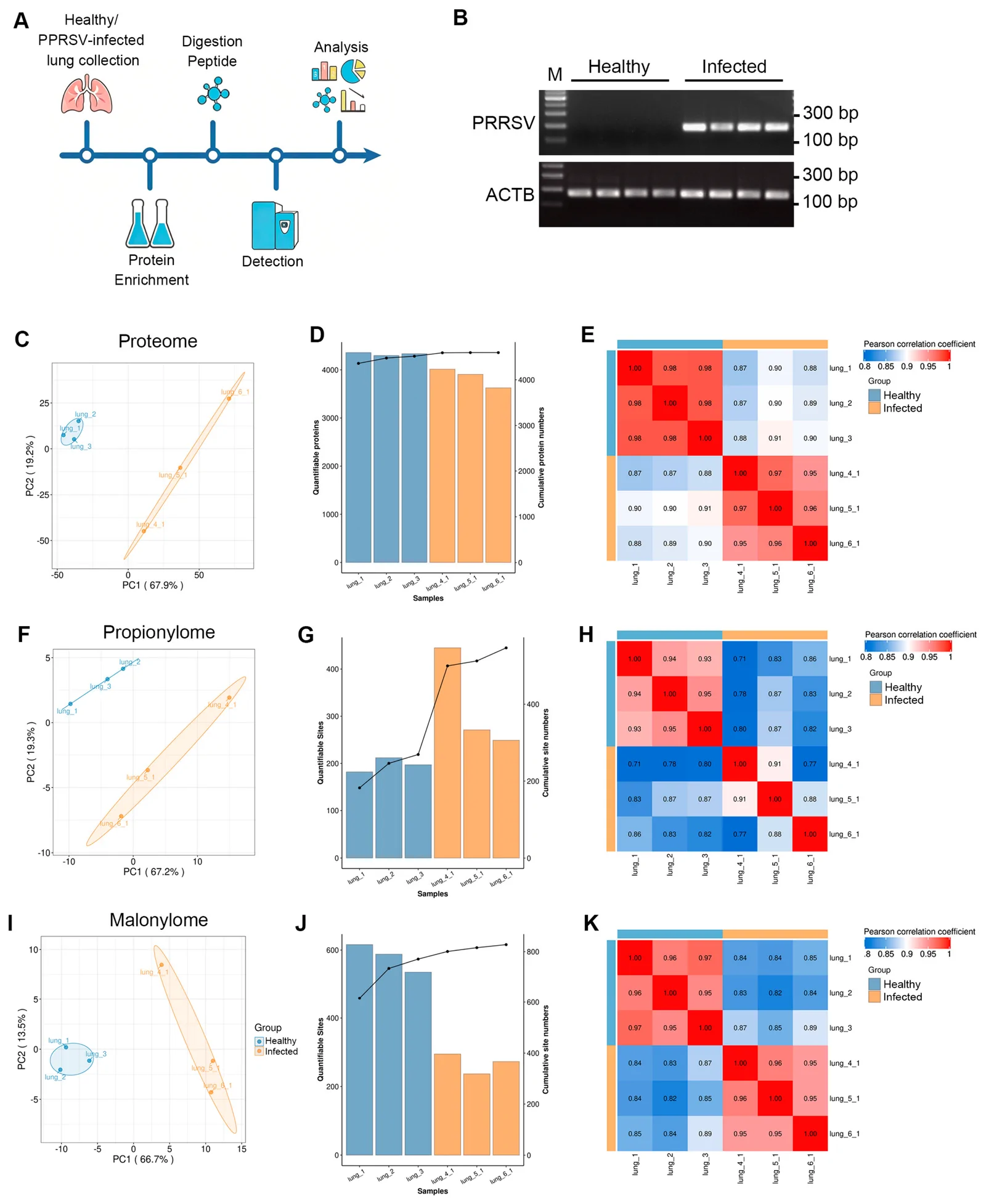
Multi-omics profiling of porcine lung tissues in response to PRRSV infection. (**A**) Schematic workflow illustrating the overall experimental design and analytical procedures for the multi-proteomic analysis. (**B**) semi-quantitative RT-PCR detection of PRRSV loads in healthy and PRRSV-infected lung tissues. (**C**–**E**) Comprehensive proteomic analysis of protein expression profiles in healthy and PRRSV-infected porcine lung tissues. (**C**) Principal component analysis (PCA) of proteomic data between healthy and infected samples. (**D**) Statistical summary of the total number of proteins identified and quantified in porcine lung tissues. (**E**) Pearson correlation analysis of proteomic data among all biological samples. (**F**–**H**) Systematic proteome-wide propionylation modification analysis of healthy and PRRSV-infected porcine lung tissues. (**F**) PCA of protein propionylation modification data between healthy and PRRSV-infected lung tissue samples. (**G**) Statistical quantification of the total number of modified proteins identified in the propionylome. (**H**) Pearson correlation analysis of protein propionylation modification data among all biological samples. (**I**–**K**) Global proteome-wide malonylation modification analysis of healthy and PRRSV-infected porcine lung tissues. (**I**) PCA of protein malonylation modification data between healthy and PRRSV-infected lung tissues. (**J**) Statistical summary of the total number of modified proteins. (**K**) Pearson correlation analysis of protein malonylation modification data among all biological samples.

To elucidate the underlying mechanisms in PRRSV-infected porcine lungs, we conducted three complementary omics analyses: quantitative proteomics, propionylomics, and malonylomics (Figure 1A). Firstly, the healthy and PRRSV-infected lung tissue were collected, and the infected status was identified by semi-quantitative RT-PCR. As expected, specific amplification of the PRRSV ORF7 gene was detected only in the infected group, with no signal in the healthy group (Figure 1B). Subsequently, the tissue proteins were lysed by lysis buffer, despite the quantitative proteome, and the proteins were enriched with anti-propionylation or anti-malonylation antibody. Then, the proteins were digested into peptide by trypsin and identified by LC-MS/MS. The proteome and post-translational modification profiles exhibited marked differences between healthy and PRRSV-infected lungs (Figure 1C–K). Specifically, lungs infected with PRRSV express fewer proteins (Figure 1D) and fewer malonylated proteins (Figure 1J); however, they have more propionylated proteins (Figure 1G). These results indicate that PRRSV infection would change the protein and post-translational modification patterns in the porcine lungs.

### 3.2. PRRSV Infection Triggers Broad Proteome Suppression and Opposite Remodeling of Propionylation and Malonylation

**Figure 2:**
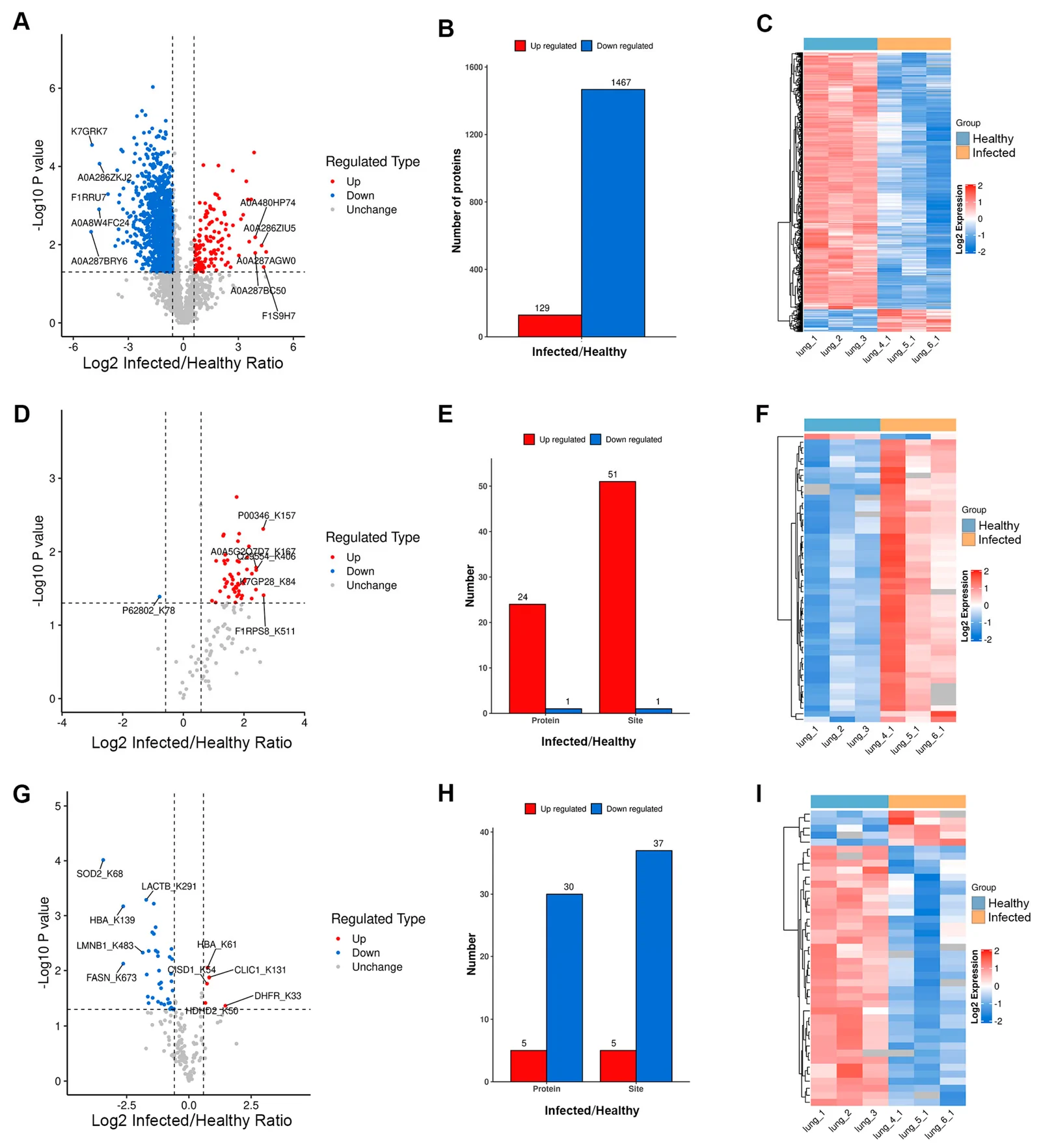
Differential expression analysis based on multi-proteomic data. (**A**) Volcano plot visualizing the distribution of differentially expressed proteins between healthy and infected groups. (**B**) Statistics for the count of differentially expressed proteins. (**C**) Heatmap exhibiting the expression patterns of all differentially expressed proteins. (**D**) Volcano plot visualizing the distribution of proteins carrying differential propionylation modification. (**E**) Statistics for the count of proteins with differential propionylation modification. (**F**) Heatmap exhibiting the modification levels of proteins with differential propionylation modification. (**G**) Volcano plot visualizing the distribution of proteins carrying differential malonylation modification. (**H**) Statistics for the count of proteins with differential malonylation modification. (**I**) Heatmap exhibiting the modification levels of proteins with differential malonylation modification.

Our quantitative proteomic and PTM analyses revealed extensive changes in both host protein expression and acyl modification profiles after infection. Quantitative proteomic profiling demonstrated a massive suppression of host protein expression, with 1467 proteins significantly down-regulated, far surpassing the 129 proteins that were up-regulated (Figure 2A–C). In addition, our differential analysis identified 25 propionylated proteins with 52 sites and 35 malonylated proteins with 42 sites that were significantly altered (Figure 2D–I). Propionylation was predominantly up-regulated, with 51 sites in 24 proteins increased and only a single site down-regulated (Figure 2D–F). In contrast, malonylation exhibited an inverse pattern, characterized by widespread down-regulation affecting 37 sites in 30 proteins, while only 5 sites in 5 proteins were up-regulated (Figure 2G–I). This divergent remodeling of the propionylome and malonylome occurred against a backdrop of extensive host proteome reprogramming.

### 3.3. Protein–Protein Interaction Network and Hub Protein Analysis

**Figure 3:**
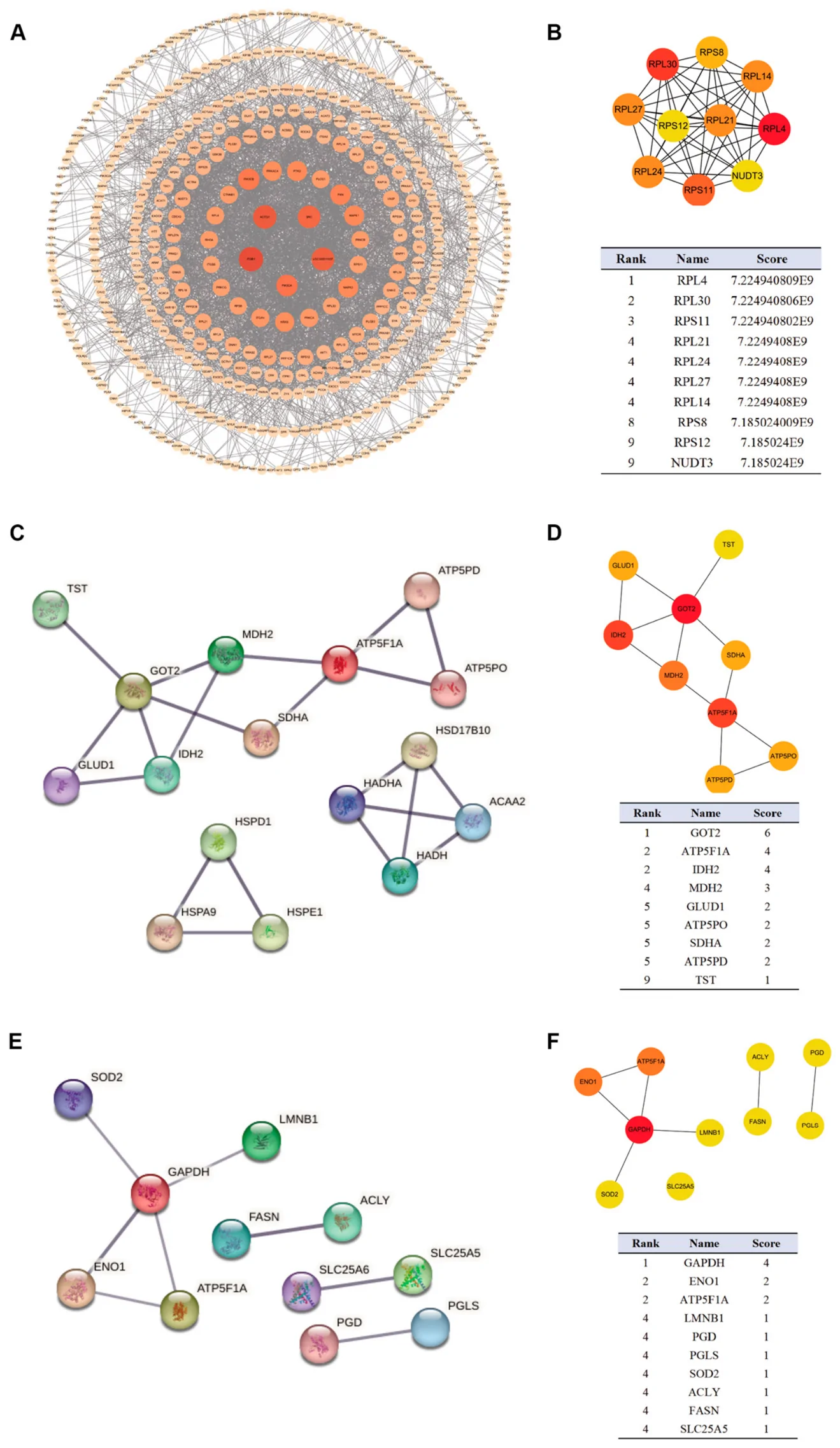
Protein–protein interaction (PPI) network analysis of quantitative proteomics and lysine acyl-modified proteomics. (**A**,**B**) PPI networks derived from the quantitative proteomics dataset. (**A**) The full network of overlapping differentially expressed proteins from the infected and healthy comparison. (**B**) The subnetwork of the top 10 hub genes identified from (**A**). (**C**,**D**) PPI networks derived from the lysine propionylome. (**C**) The full network of enriched propionylated proteins from the infected and healthy comparison. (**D**) The subnetwork of the top 10 hub propionylated proteins. (**E**,**F**) PPI networks derived from the lysine malonylome. (**E**) The full network of enriched malonylated proteins from the infected and healthy comparison. (**F**) The subnetwork of the top hub malonylated proteins, as listed. All PPI networks were constructed using the STRING database and visualized with Cytoscape software (v3.7.0). The top hub genes in (**B**,**D**,**F**) were identified and ranked using the CytoHubba plugin based on Maximal Clique Centrality algorithm.

Protein–protein interaction (PPI) networks were constructed for the proteome, propionylome, and malonylome. We systematically examined interactions between key differentially expressed proteins and proteins harboring differentially modified peptides across the proteome, propionylome, and malonylome. In the quantitative proteome PPI network (Figure 3A), a total of 460 nodes were identified, comprising 16 up-regulated and 444 down-regulated proteins. Network analysis revealed 10 core hub proteins, including 9 ribosomal proteins (RPL4, RPL30, RPS11, RPL21, RPL24, RPL27, RPL14, RPS8, RPS12) and NUDT3, an enzyme involved in mRNA metabolism (Figure 3B). These hub proteins maintained high network centrality despite widespread suppression of global protein expression, suggesting that PRRSV infection may create a favorable intracellular environment for viral replication by modulating host protein synthesis machinery and mRNA metabolic processes.

For the propionylome, the PPI network contained 17 protein nodes, all of which were upregulated. Key hub proteins identified included GOT2, ATP5F1A, IDH2, MDH2, GLUD1, ATP5PO, SDHA, ATP5PD, and TST (Figure 3C,D). Notably, most of these proteins represent core components of essential energy metabolism pathways, including the tricarboxylic acid cycle, electron transport chain, and ATP synthesis. The significant alterations in propionylation levels of these metabolic enzymes indicate that PRRSV may reprogram the host cell metabolic network through direct modification of energy metabolism enzymes, potentially enhancing biosynthetic precursor supply and ATP production to meet the substantial energy and material demands of viral replication.

In the malonylome PPI network, 11 nodes were identified, all showing down-regulation. The most prominent proteins included GAPDH, ENO1, ATP5F1A, LMNB1, PGD, PGLS, SOD2, ACLY, FASN, and SLC25A5 (Figure 3E,F). These proteins are primarily involved in fatty acid metabolism and energy metabolism, indicating that PRRSV infection is associated with extensive metabolic reprogramming, particularly affecting lipid and energy metabolism. The observed alterations in malonylation modification may represent a viral strategy to modulate host metabolism, thereby facilitating viral replication by ensuring adequate lipid supply for envelope formation and sufficient energy production.

### 3.4. Functional Enrichment Analysis of the Differentially Expressed Protein/Sites

**Figure 4:**
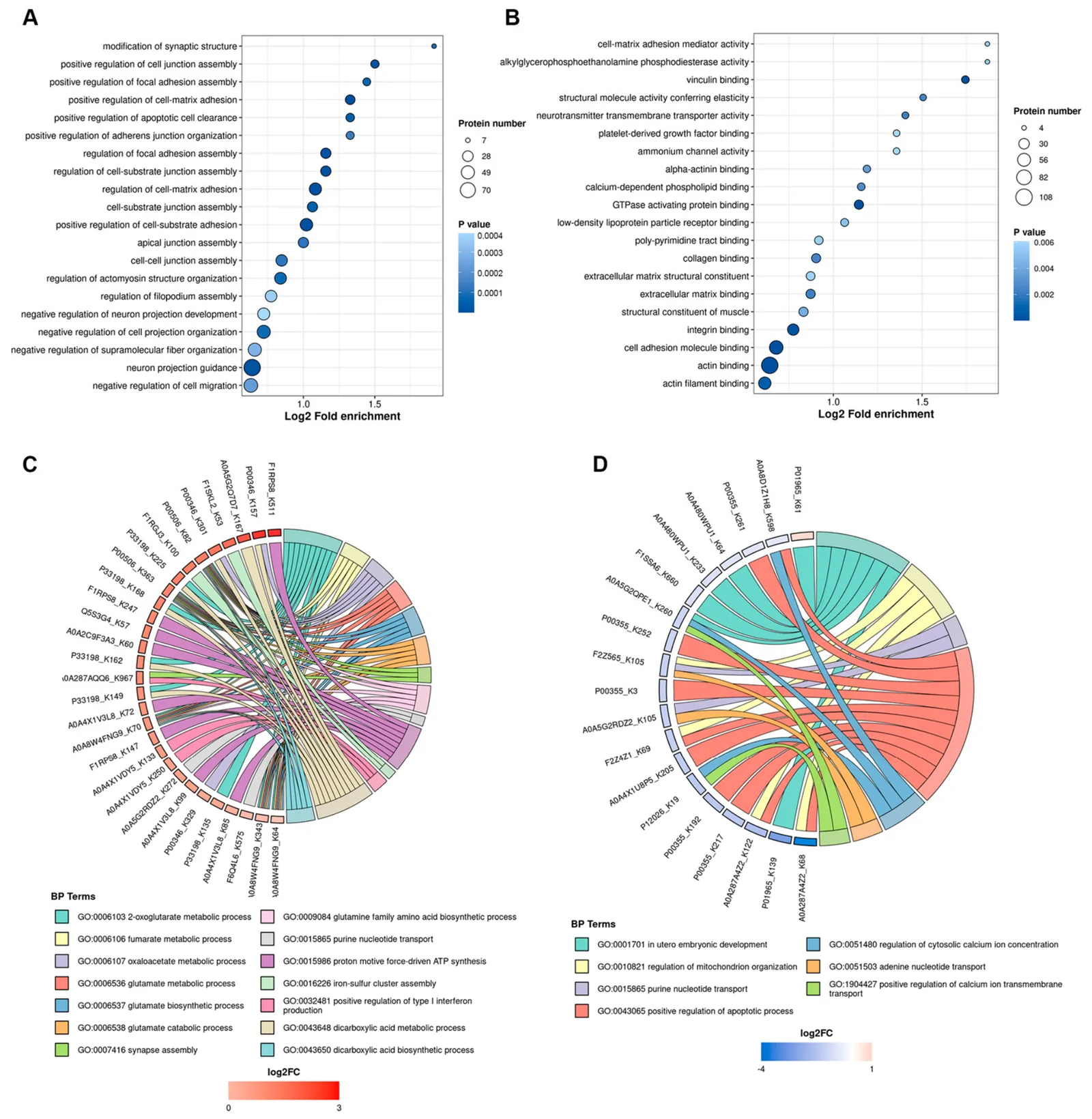
Functional enrichment analysis of differential proteins. (**A**) Biological process enrichment analysis for differentially expressed proteins. (**B**) Molecular function enrichment analysis for differentially expressed proteins. (**C**) Biological process enrichment analysis for proteins with differential propionylation modification. (**D**) Biological process enrichment analysis for proteins with differential malonylation modification.

GO enrichment of global DEPs showed that downregulated proteins were significantly enriched in biological processes related to cell adhesion, extracellular matrix (ECM) organization and actin cytoskeleton regulation (Figure 4A), with molecular functions concentrated in ECM receptor binding, actin binding and cell adhesion molecule activity (Figure 4B). These results indicate that PRRSV infection disrupts pulmonary cell–cell junctions and tissue structural integrity, consistent with the respiratory barrier impairment observed in PRRS.

For the propionylome, differentially propionylated proteins were predominantly enriched in mitochondrial metabolic processes, including the tricarboxylic acid cycle, oxidative phosphorylation and ATP biosynthesis (Figure 4C), confirming the targeted regulation of mitochondrial energy metabolism by propionylation. For the malonylome, differentially malonylated proteins were enriched in carboxylic acid metabolism, fatty acid biosynthesis and apoptotic process regulation (Figure 4D), suggesting a potential role of malonylation in modulating lipid metabolism and cell fate during PRRSV infection.

In summary, PRRSV infection disturbs the propionylation and malonylation modification levels of proteins, thereby triggering dysregulation of energy metabolism and lipid metabolism in porcine lung tissue cells and disrupting intracellular energy conversion and normal physiological functions.

### 3.5. Integrated Analysis of Three Omics Identifies the Main Pathway of PRRSV Infection

**Figure 5:**
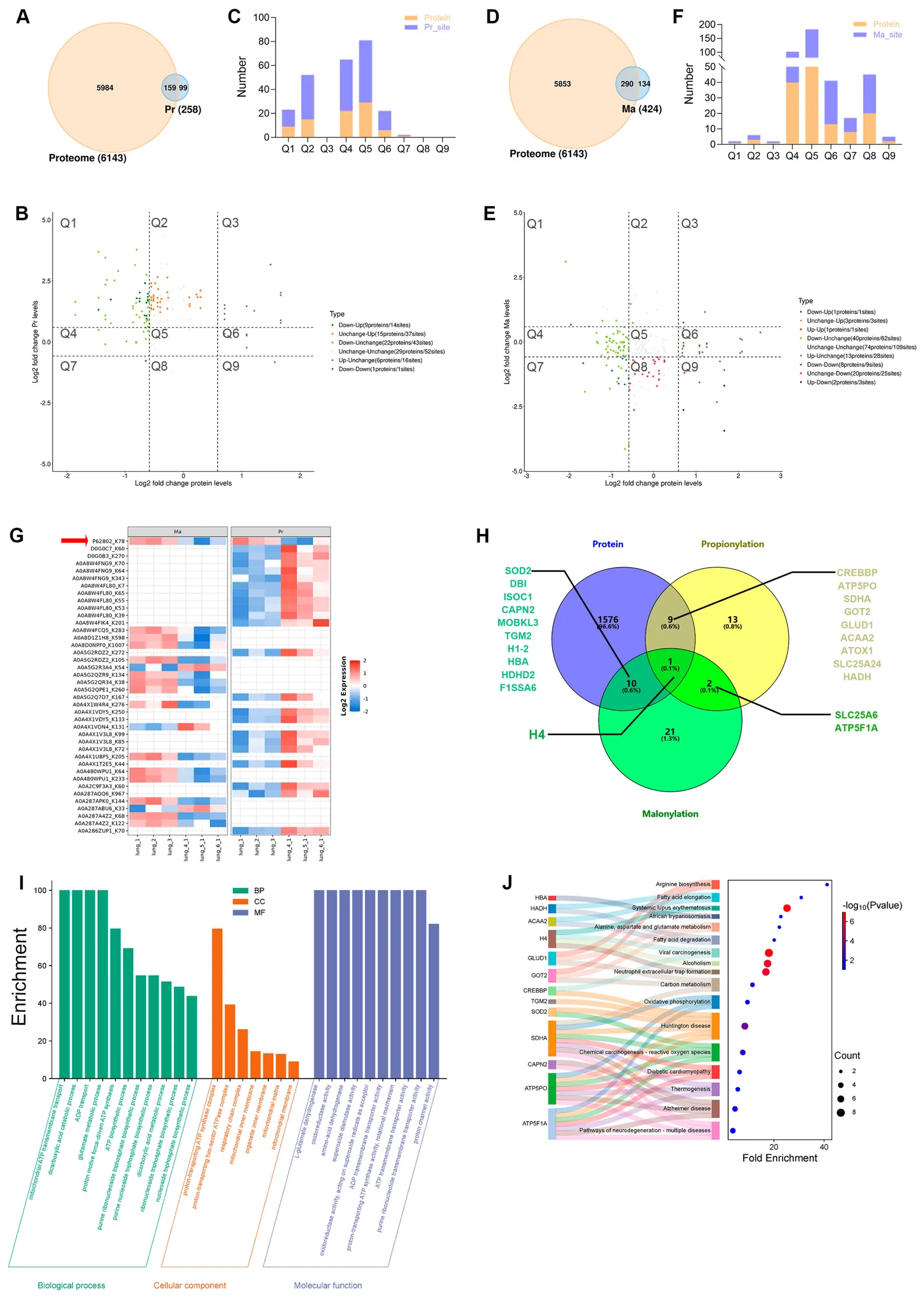
Integrative analysis of multi-proteomics analysis. (**A**) Overlap analysis of proteins identified from the proteome and propionylome. (**B**) Nine-quadrant plot of differential proteins derived from proteome and propionylome data. (**C**) Statistics of significantly altered proteins/modification sites distributed in different quadrants shown in panel (**B**). (**D**) Overlap analysis of proteins identified from the proteome and malonylome. (**E**) Nine-quadrant plot of differential proteins derived from proteome and malonylome data. (**F**) Statistics of significantly altered proteins/modification sites distributed in different quadrants shown in panel (**E**). (**G**) Heatmap displaying differentially modified sites of proteins with differential propionylation and malonylation. (**H**) Overlap analysis of differential proteins obtained from the three omics datasets. (**I**) Functional enrichment analysis of combined differential proteins, including biological process, cellular component and molecular function categories. (**J**) Sankey diagram illustrating KEGG enrichment results of combined differential proteins.

First, we integrated data from proteome and propionylome profiling. A total of 159 proteins were jointly identified by the two omics datasets (Figure 5A). Comparative analysis of differentially expressed proteins (DEPs) upon PRRSV infection at both proteomic and propionylation levels revealed that most DEPs were distributed in the Q1–Q2 quadrants (Figure 5B,C), indicating a positive correlation between low protein abundance and elevated propionylation modification levels. Subsequently, we performed integrative analysis of the proteome and malonylome. The two datasets jointly captured 290 proteins (Figure 5D). Nevertheless, only a small subset of proteins exhibited concurrent significant alterations in total protein abundance and malonylation levels (Figure 5E,F), demonstrating that changes in total protein expression are largely independent of malonyl modification dynamics. Next, we compared differentially modified proteins and their corresponding modification sites between propionylation and malonylome datasets. Only three overlapping differentially modified proteins were identified, with merely one shared modification site (Figure 5G). This result suggested that PRRSV infection induces opposite alteration trends in propionylation and malonylation levels; these two post-translational modifications (PTMs) do not exert antagonistic effects but instead regulate biological processes via distinct signaling pathways.

Integrated analysis of DEPs across all three omics datasets identified histone H4 as a candidate with significant differential expression. Notably, the critical modification site on histone H4 undergoes competitive propionylation and malonylation under PRRSV challenge (Figure 5H), which may modulate histone H4 function. However, this expression pattern needs to be validated by site-directed mutagenesis. Subsequently, GO analysis shows that the three-omics differentially expressed proteins were energy metabolic-related proteins; they participate in ADP and ATP transport and energy support (Figure 5J), which may result in lung pathology. In conclusion, these findings illustrate that PRRSV infection caused a multi-tiered regulatory model: at the global pathway level, propionylation and malonylation regulate distinct biological processes in a non-antagonistic manner; at the local site level, competitive modification of shared residues such as histone H4 adds a finer layer of regulatory specificity.

## 4. Discussion

Post-translational modifications (PTMs) are central to PRRSV–host interplay, and the virus hijacks multiple canonical PTM pathways (e.g., phosphorylation, ubiquitination, lactylation) to support replication and immune evasion [34,35,36,37,38,39,40,41,42,43,,44,45]. However, the roles of metabolism-sensitive lysine acyl modifications, particularly propionylation and malonylation, remain entirely uncharacterized during PRRSV infection. Here, we present a foundational landscape study providing the first integrated map of these two acylations in PRRSV-infected porcine lung tissue.

We observed a global reduction in host protein abundance upon PRRSV infection, with 1467 proteins significantly downregulated versus only 129 upregulated, consistent with prior reports of PRRSV-induced host shutoff in alveolar macrophages [22,31]. The downregulated proteins were enriched in cell adhesion and ECM organization, which may explain the impaired pulmonary barrier function and tissue damage observed in PRRSV-infected pigs. Strikingly, against this backdrop of widespread protein suppression, we detected divergent, highly specific reprogramming of the two PTMs independent of de novo protein synthesis: lysine propionylation was predominantly upregulated, whereas malonylation was broadly downregulated. This opposing pattern indicates that PRRSV does not merely induce global host suppression but actively fine-tunes critical pathways via PTMs—a strategy that allows rapid metabolic and cellular reprogramming even when host protein production is compromised.

Functional and network analyses revealed that hyper-propionylated proteins are strongly enriched in mitochondrial metabolic pathways, with core tricarboxylic acid cycle enzymes (GOT2, IDH2, MDH2) and oxidative phosphorylation components (ATP5F1A, ATP5PO) identified as key hubs. Lysine propionylation modulates enzyme activity by altering protein charge and conformation, and its upregulation on these metabolic hubs may contribute to enhanced mitochondrial energy production and biosynthetic flux, though functional validation is required. This provides a mechanistic explanation for previous observations that PRRSV rewires cellular metabolism to generate an energy-sufficient, lactate-rich environment for replication [44,46]: rather than upregulating metabolic enzyme expression (blocked by global host shutoff), the virus activates existing metabolic machinery via propionylation to secure ATP and biosynthetic precursors. This PTM-mediated metabolic rewiring represents a convergent viral strategy, as evidenced by KSHV encoding a viral propionyltransferase to modulate host function and evade immunity [29].

In contrast, malonylation was broadly downregulated during PRRSV infection. Hypo-malonylated proteins were enriched in lipid metabolism and cell fate regulation, including enzymes involved in fatty acid synthesis (ACLY, FASN) and glycolysis (GAPDH, ENO1). Malonylation is generally known to inhibit metabolic enzyme activity by competing with cofactor binding; its downregulation may enhance glycolytic and lipid biosynthetic flux to supply energy and membrane components for viral particle assembly. Additionally, the downregulation of malonylation on apoptosis-associated proteins may modulate infected cell fate, potentially associated with viral persistence and immune evasion, though this requires further functional validation. The divergent regulation of propionylation and malonylation may also be linked to changes in intracellular acyl-CoA pools: PRRSV-induced metabolic reprogramming may increase propionyl-CoA levels while depleting malonyl-CoA for lipid biosynthesis, driving the opposite modification patterns observed here.

Several limitations of this study should be noted. These findings are based on tissue-level omics profiling, and the functional consequences of specific modification sites remain to be validated via site-directed mutagenesis and in vitro infection assays. The regulatory enzymes (acyltransferases/deacylases) and upstream metabolic signals driving the divergent acylation changes were not identified. In addition, the sample size of this research needs to be expanded, cell-type resolution analysis could be utilized to identify which cell type is the major response cell type, and more sample collection time points could be set as we can clarify whether the PTM alteration is the outcome or the acute response to PRRSV infection. Future research could be improved by addressing these limitations.

In summary, this study provides the integrated landscape of the host proteome, propionylome and malonylome in PRRSV-infected porcine lung tissue. We demonstrate that PRRSV induces robust global suppression of host protein expression, disrupting cell adhesion and structural pathways to impair pulmonary barrier function. Critically, we identify a distinct opposing reprogramming of lysine propionylation and malonylation that operates independently of global protein expression changes: upregulated propionylation targets mitochondrial metabolic enzymes to support viral energy acquisition, while downregulated malonylation may modulate lipid metabolism and cell fate to facilitate viral persistence. This work reveals the PTM-centric host–virus interaction pathway and provides candidate molecular targets for future functional validation and antiviral development.

## Data Availability

The data presented in this study are available on request from the corresponding author.

## Abbreviations

The following abbreviations are used in this manuscript:

PRRSV	Porcine reproductive and respiratory syndrome virus
PRRS	Porcine reproductive and respiratory syndrome
PTM(s)	Post-translational modification(s)
GO	Gene Ontology
KEGG	Kyoto Encyclopedia of Genes and Genomes
PPI	Protein–protein interaction
PCA	Principal component analysis
RT-PCR	Reverse transcription–polymerase chain reaction
ECM	Extracellular matrix
